# The potential of galactomannan from Caesalpinia ferrea on erosive dentin wear reduction in vitro

**DOI:** 10.1590/0103-6440202305508

**Published:** 2023-12-22

**Authors:** Cibele Sales Rabelo, Juliana Marinho Ramos de Oliveira, Isabelly de Carvalho Leal, Flávia de Miranda Leão Leite Costa, Nágila Maria Pontes Silva Ricardo, Vanara Florêncio Passos

**Affiliations:** 1 Department of Clinical Dentistry, School of Pharmacy, Dentistry and Nursing, Federal University of Ceará, Fortaleza, Ceará, Brazil.; 2 Department of Education, Federal Institute of Ceará, Horizonte, CE, Brazil.; 3 Department of Organic and Inorganic Chemistry, Federal University of Ceará, Fortaleza, Ceará, Brazil.; 4 Department of Restorative Dentistry, School of Pharmacy, Dentistry and Nursing, Federal University of Ceará, Fortaleza, Ceará, Brazil.

**Keywords:** erosion, dentin, gels, Caesalpinia

## Abstract

Gels containing juca seed galactomannan (JSG) were evaluated for their potential to prevent the progression of dentin erosive wear in an in vitro study with four experimental groups (n = 9). The treatments included distilled water (DW), 0.05% stannous fluoride (121 ppm F), and 0.5% or 1% JSG. The specimens underwent a cycle (3 times/day) consisting of immersion in 1% citric acid (5 minutes), treatment (5 minutes), and artificial saliva exposure (2 hours/overnight) for 5 days. Surface changes were assessed using mechanical profilometry (wear), scanning electron microscopy (SEM), and energy-dispersive X-ray spectroscopy (EDS). The data were analyzed using ANOVA followed by Tukey's post-test (p < 0.05). The negative control group exhibited the highest wear (6.0 µm ± 3.5), significantly differing from the group treated with 0.05% stannous fluoride gel (p = 0.007), which showed less dentin loss. The groups treated with 0.5% and 1% JSG showed results similar to the negative control (p = 0.661; p = 0.212, respectively) and the stannous fluoride group (p = 0.103; p = 0.379, respectively). In the SEM images, the specimen treated with stannous fluoride showed obliterated tubules, while the JSG gels formed crystals on the dentin surface, as confirmed by the presence of oxygen and calcium in the EDS analysis. Although the JSG gels showed similar results to the stannous fluoride, did not exhibit superior efficacy at the tested concentrations.

## Resumo

Géis contendo galactomanana da semente de jucá (GSJ) foram avaliados quanto ao seu potencial na prevenção da progressão do desgaste erosivo dentinário em um estudo in vitro com quatro grupos experimentais (n = 9). Os tratamentos incluíram água destilada (AD), 0,05% de fluoreto de estanho (121 ppm F) e 0,5% ou 1% de GSJ. Os espécimes passaram por um ciclo (3 vezes ao dia) que consistia em imersão em ácido cítrico 1% (5 minutos), tratamento (5 minutos) e exposição a saliva artificial (2 horas/durante a noite) por 5 dias. As alterações de superfície foram avaliadas utilizando perfilometria mecânica (desgaste), microscopia eletrônica de varredura (MEV) e espectroscopia de raios-X por dispersão de energia (EDS). Os dados foram analisados por ANOVA, seguida do pós-teste de Tukey (p < 0,05). O grupo controle negativo apresentou o maior desgaste (6,0 µm ± 3,5), diferindo significativamente do grupo tratado com gel de fluoreto de estanho a 0,05% (p = 0,007), que apresentou menor perda de dentina. Os grupos tratados com 0,5% e 1% de GSJ apresentaram resultados semelhantes ao grupo controle negativo (p = 0,661; p = 0,212, respectivamente) e ao grupo de fluoreto de estanho (p = 0,103; p = 0,379, respectivamente). Nas imagens de MEV, o espécime tratado com fluoreto de estanho mostrou túbulos obliterados, enquanto os géis GSJ formaram cristais na superfície da dentina, como confirmado pela presença de oxigênio e cálcio na análise de EDS. Embora os géis GSJ tenham apresentado resultados semelhantes ao fluoreto de estanho, eles não demonstraram eficácia superior nas concentrações testadas.

## Introduction

Lifestyle changes and dietary habits play a crucial role in dental erosion ^1^, ^2^. Over the past few decades, there has been a significant rise in the global consumption of energy drinks, carbonated beverages, fruit juices, and citrus fruits. These items contain dietary acids, which are considered external factors that contribute to erosive tooth wear (ETW) ^3^, ^4^. When the teeth are frequently exposed to acids, minerals are lost from the tooth surface, resulting in softening. This erosive process is often associated with mechanical factors, such as abrasion caused by oral habits, and attrition due to tooth-to-tooth contact, which further contribute to the progression of dental wear ^5^, ^6^.

Clinically, repeated exposure to acidic substances results in dental enamel wear, leading to the exposure of the dentin ^6^. As erosive tooth wear progresses from moderate to severe levels, dentin becomes increasingly exposed. This erosive effect exposes the organic matrix, which can be degraded both mechanically and chemically ^7^. Additionally, the presence of enzymes such as matrix metalloproteinases (MMPs) can degrade this matrix, contributing to further tooth wear. Consequently, the identification and development of products that can effectively inhibit MMPs play a critical role in reducing mineral loss in dentin ^8^.

The most common treatments for tooth erosion involve the use of fluoride-containing compounds, which can result in the formation of calcium fluoride or metal-rich precipitates ^9^, ^10^. It has been reported that stannous fluoride (SnF_2_) is an excellent compound that significantly enhances the resistance of the dental surface to acids. ^10^. However, natural products are increasingly being explored as promising sources for new therapeutic agents due to their renewability and low toxicity ^11^-^13^.

An agarose hydrogel, a natural polysaccharide, has shown benefits in dentin remineralization and dental tubule occlusion ^14^. Several plant species contain hydrophilic polysaccharides called galactomannans in their seed endosperm. Caesalpinia ferrea, commonly known as juca, is a plant commonly found in the northern and northeastern regions of Brazil. It possesses a polysaccharide with high intrinsic viscosity, forming highly viscous solutions even at low concentrations ^15^. Additionally, C. ferrea exhibits a rich phenolic composition with antioxidant potential and enzymatic inhibitory activity ^16^, ^17^.

Therefore, considering the properties of juca, it is important to investigate its potential as a strategy for reducing dentin mineral loss during erosive challenges. However, to the best of the authors' knowledge, there have been no studies evaluating the effects of juca seed galactomannan (JSG) gels in preventing the progression of dental erosion. Therefore, this study aimed to assess the effects of JSG gels on reducing the erosive process of extrinsic origin in human root dentin. The null hypothesis stated that JSG gels would not have the ability to reduce dentin erosion in vitro.

## Materials and methods

### Ethical aspects

This study was submitted to and approved by the Research Ethics Committee of the Federal University of Ceará (protocol #2.611.040). Sound human third molars were used with the patient's consent. The teeth were stored in a 0.01% (w/v) thymol solution at 4°C.

### Experimental design

It was a randomized, in vitro study. The experiment consisted of four experimental groups (n=9) with the following treatments: distilled water (DW), 0.5% juca seed galactomannan (JSG) gel, 1% JSG gel, and 0.05% stannous fluoride gel (121 ppm F) (Table 1). The erosive effect was assessed through quantitative measurement of tissue loss using a profilometer and qualitative analysis using SEM.

**Table 1:** Description of the gels used.

Products	Composition	pH
Stannous fluoride gel	0.05 % stannous fluoride, 20% PLURONIC F127® and distilled water	4.0
Juca seed galactomannan gel 0.5%	0.5% juca seed galactomannan and distilled water	7.1
Juca seed galactomannan gel 1%	1% juca seed galactomannan and distilled water.	7.0

### Sample size calculation

Based on the study by Silveira et al. ^13^, which observed that dentin loss in a group treated with anacardic acid was significantly lower than the control group treated with sodium fluoride (1.97±1.02 vs 3.93±1.54), it was estimated that seven specimens per study group would be necessary to obtain a sample that represents the alternative hypothesis of this study with 80% statistical power and 95% confidence (Student's t-test). Considering the possibility of sample loss, an additional 20% was added to this sample, resulting in a total of nine specimens per group.

### Specimen preparation

Root dentin specimens (4 × 4 × 2 mm) were obtained from human third molars using a hard tissue-cutting machine (IsoMetTM Low-Speed Saw Buehler, Lake Bluff, IL, USA). The specimens were then fixed onto acrylic resin discs. The dentin blocks were planned and polished according to the methods described in a previous study ^11^. After polishing, the specimens were sonicated in distilled water for 2 minutes. To protect the reference surface, a piece of tape was applied to each side of the specimen, leaving a center area (1 × 4 mm) exposed.

All specimens were evaluated for initial hardness through a microhardness tester (FM-ARS 9000; Future Tech Corp., Tokyo, Japan) with a Knoop diamond tip. Five indentations were performed in the center of the blocks, with a load of 10 g for 5s and a distance of 100 μm.

All specimens were assessed for their initial hardness using a microhardness tester (FM-ARS 9000; Future Tech Corp., Tokyo, Japan) equipped with a Knoop diamond tip. In the center of each block, five indentations were made with a load of 10 g for 5 seconds and a spacing of 100 μm. A total of 36 specimens with an average hardness of 58.46 ± 3.29 kg/mm² were selected and randomly assigned to the experimental groups using a computer-generated randomization list created in Microsoft Excel 365.

### Isolation, purification of the JSG, and Preparation of the gels

The juca seedpods were collected at the Pici campus of the Federal University of Ceará, in Fortaleza, Ceará state, Brazil. The plant specimen was deposited in the Prisco Bezerra Herbarium at the same university, with registration number 44695.

The juca seedpods were selected, washed, and dried at 40 °C. Subsequently, the seeds were separated and soaked in distilled water at 85°C for 30 minutes. The endosperm was then extracted and subjected to a water and alcohol partition (1:4, v/v) at 60°C for 20 minutes. To extract the galactomannan, 10 grams of the depigmented endosperm were lyophilized and dissolved in 900 ml of water. The mixture was heated on a hot plate at 75°C for 4 hours, filtered, and the residue was discarded. For galactomannan purification, 97% PA ethanol (1:3, v/v) was added under stirring for 4 hours, along with NaCl (2.0 g/mol). The resulting mixture underwent three cycles of precipitation using 99% ethanol (1:1, v/v), followed by filtration and centrifugation. The gelatinous precipitate was then dried through lyophilization. ^16^. The JSG gels were prepared by dissolving the galactomannan in distilled water at 60°C with magnetic stirring.

The copolymer PLURONIC F127® (Sigma Chemical Co., St. Louis, MO, USA) was utilized to prepare a gel containing stannous fluoride at a concentration of 20%. The distilled water was cooled in an ice bath while being agitated continuously with the addition of the copolymer PLURONIC F127® until it completely dissolved.

### Erosive cycling model

The study involved a cyclic experiment that was repeated for five consecutive days, comprising three main steps: an erosive challenge, treatment with gels, and remineralization using artificial saliva. Each specimen was exposed to a 0.05 M citric acid solution (pH 1.3) for 5 minutes. Following the erosive challenge, the specimens were treated with the respective gels for 5 minutes. Subsequently, the blocks were rinsed with distilled water and immersed in artificial saliva (1.5 mM Ca; 0.9 mM PO4; 150 mM KCl; 0.1M Tris buffer) for 2 hours (11). This cycle of acid exposure, gel treatment, and remineralization was repeated three times a day for five consecutive days. To ensure optimal conditions, the specimens were stored overnight at 37°C in artificial saliva.

### Surface wear evaluation

To measure tooth wear, a Hommel Tester T1000 mechanical profilometer (Hommelwerke GmbH, Germany) was used after the completion of the experimental period. The height difference between the reference surface and the treated area was assessed to quantify the extent of tooth wear. Before analysis, the tape covering the untreated area was removed, exposing the surface for evaluation. Within each sample, three measurements were taken at 100 µm intervals over a 1.5 mm distance. The average of these measurements was calculated for each sample, providing an assessment of tooth wear ^18^.

### SEM and EDS evaluations of the dentin surfaces

Two dentin samples from each group were subjected to scanning electron microscopy (SEM). They were immersed in a 2.5% glutaraldehyde fixative solution in a 0.1 mol/L sodium cacodylate buffer for 24 hours and then washed with the same buffer. Subsequently, the samples were dehydrated using ethanol solutions with increasing concentrations and dried at room temperature for 24 hours in a desiccator ^19^. The dried specimens were fixed onto metal stubs and coated with a layer of gold using a metallizer (Hammer VI - Sputtering System, Anatech Ltda, Alexandria, USA).

The samples were analyzed using the SEM Quanta FEG 450 (FEI Company, Oregon, USA). Subsequently, an EDS analysis was conducted to examine the chemical composition of the dentin surface. The acceleration voltage was set at 20 kV, and a magnification of 5000x was used.

### Statistical analysis

The mean and standard deviation data were analyzed using the SPSS 22.0 software for Windows (SPSS Inc., Chicago, IL, USA). Preliminary Kolmogorov-Smirnov tests for normality were conducted. Based on the normal distribution observed in the evaluated data, parametric analysis of variance (ANOVA) tests, one-factor, were performed. Post-hoc comparisons were conducted using the Tukey test for the wear data, with a significance level set at 5%.

## Results

The wear values (µm) of the root dentin (Table 2) revealed a statistically significant difference between the groups (p = 0.011). The negative control group (distilled water) exhibited the highest wear pattern (6.0 µm ± 3.5), which was significantly different from the group treated with 0.5% stannous fluoride gel (p = 0.007), as it showed the lowest dentin loss. The groups treated with 0.5% and 1% JSG gel showed similar results to the negative control (p = 0.661; p = 0.212, respectively) and stannous fluoride (p = 0.103; p = 0.379, respectively). However, there was no significant difference in comparison to the galactomannan gel concentration (p = 0.850).

**Table 2:** Mean and standard deviation of dentin surface loss in µm.

Groups	Surface loss (µm)
Distilled water	5.97 (± 3.56)a
Stannous fluoride gel	2.39 (± 0.60)b
Juca seed galactomannan gel 0.5%	4.80 (± 1.66)ab
Juca seed galactomannan gel 1%	4.00 (± 1.73)ab

Different letters indicate significant differences among groups (p < 0.05).

**Figure 1:**
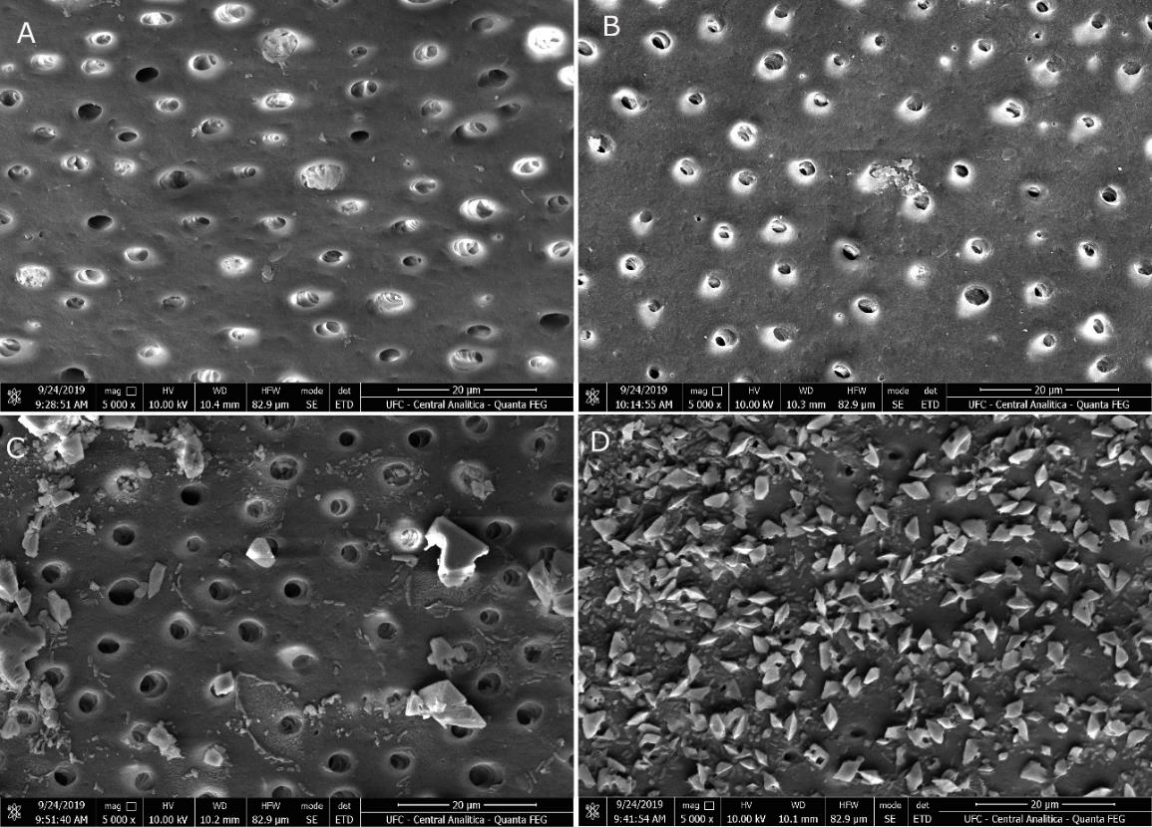
Representative SEM micrograph (x5000) of erosion-treated area. Distilled water (A), stannous fluoride (B), JSG 0.5% (C), and JSG 1% (D).

**Figure 2:**
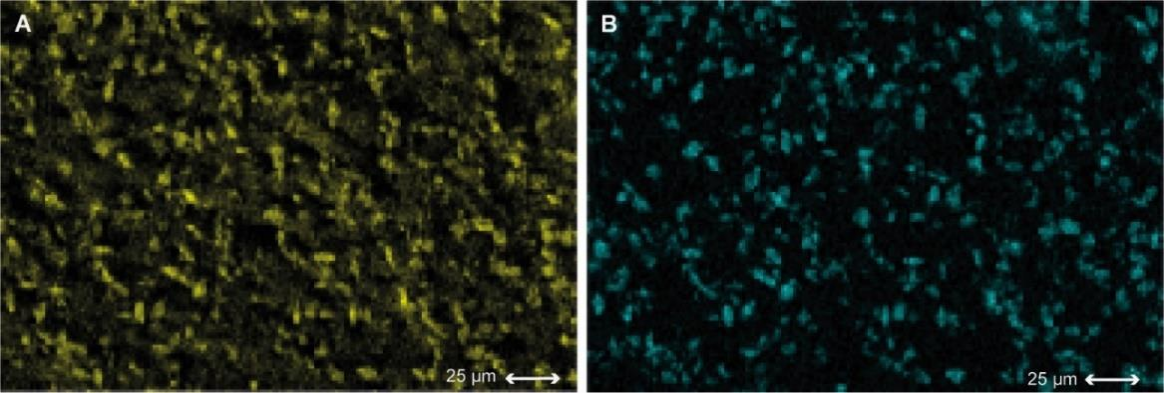
EDS images of the treated area with JSG 1%. The yellow color indicates the presence of oxygen (A) and the blue color indicates the presence of calcium (B) in the dentin surface.

The SEM images reveal that the negative control group (Figure 1a) exhibits wider dentinal tubules. In contrast, the specimen treated with stannous fluoride (Figure 1b) shows more obliterated tubules. The specimen treated with JSG at a concentration of 0.5% (Figure 1c) displays the formation of poorly defined crystals, whereas the one treated with JSG at 1% exhibits a higher quantity of crystals with a more distinct shape (Figure 1d). Energy dispersive X-ray spectroscopy (EDS) analysis enables the detection of calcium and oxygen on the surface of the dentin specimen treated with JSG 1% (Figure 2).

## Discussion

According to our results, the null hypothesis stating that JSG gels do not significantly prevent the progression of dental erosion was accepted. In terms of wear analysis, the group treated with stannous fluoride gel showed a significant difference compared to the negative control group, exhibiting less dentin loss. Both concentrations of JSG did not demonstrate statistical differences compared to the negative control. However, they were also similar to the positive control (stannous fluoride).

In this in vitro study, surface profilometry was employed to detect surface loss in dentin specimens. This technique quantifies surface loss by comparing it to an untreated reference area. It has been utilized in numerous studies and is considered a reliable method for evaluating surface loss in specimens subjected to significant erosion. ^11^, ^13^, ^18^.

Previous studies have demonstrated positive outcomes in terms of reducing the progression of dental erosion using stannous fluoride. ^9^, ^10^. Stannous fluoride functions by creating a protective barrier on the tooth surface, facilitated by the strong affinity between stannous ions and hydroxyapatite ^10^. The findings of the current study further support the beneficial effects of SnF_2_ gel in preventing erosion caused by citric acid, as it was observed to decrease wear compared to the negative control.

The present study examined a gel formulation based on a natural product intended for in-office application. Previous studies have also investigated gels containing natural products ^20^, ^21^. In a study simulating extrinsic erosion using bovine dentin blocks, a Neem gel (10% Neem extract) did not show a significant difference compared to the negative control in reducing dentin loss. Only when combined with sodium fluoride gel was there a reduction in dentin loss ^20^. Similarly, in the present study, JSG also did not demonstrate a significant difference. However, the gels in this study were prepared using isolated galactomannan. It is possible that incorporating another active ingredient could provide additional benefits. In another study utilizing bovine dentin blocks, a 10% cranberry extract gel was effective in reducing dentin loss ^21^. The concentrations of JSG used in this study were 0.5% and 1%, which are much lower compared to the concentrations employed in the previously mentioned studies. Therefore, it is believed that using a higher concentration of JSG may yield better results in reducing dentin loss.

SEM analysis is commonly utilized to assess changes in dentin morphology ^13^. In an in vitro study, the closure of dentinal tubules was observed using various concentrations of SnF_2_ ^22^. In the current study, SEM analysis of specimens treated with stannous fluoride revealed a reduction in tubule diameter compared to the control group, along with the occlusion of certain dentinal tubules. In the SEM images of blocks treated with JSG, crystal deposition was observed on the dentin surface in both concentrations tested, with the gel containing the higher concentration of galactomannan displaying more clearly defined crystals. To determine the composition of these crystals, an EDS analysis was performed.

EDS analysis conducts a mapping of the dentin surface, allowing for the identification of chemical elements present ^23^. A significant presence of oxygen and calcium was observed in the region where the crystals were located. Furthermore, the morphology of the crystals formed by JSG gels resembled that of calcium oxalate crystals reported in previous studies ^24^, ^25^. In one study, phytocomplexes containing oxalates were found to reduce dentin permeability through crystal deposition within the dentinal tubules ^25^. These findings suggest that JSG may play an important role in decreasing dentin permeability.

Some species of the Caesalpinia genus are well-known and extensively studied, but only a few of the polysaccharides extracted from their pods and seeds are utilized for therapeutic and commercial purposes ^17^. Although there was no significant difference observed between the concentrations of JSG gels, SEM images of the JSG 1% group showed a notable formation of well-defined crystals, indicating that this concentration warrants further investigation. Considering the morphological changes in dentin observed in the SEM images, it is plausible to consider the potential use of JSG in the treatment of dentin hypersensitivity, which is one of the complications associated with erosion-induced wear.

As this study was conducted in vitro, it is important to note that the results cannot be directly extrapolated to in vivo situations, as the oral cavity's natural protective factors are absent. One limitation of the study is the use of artificial saliva, which lacks the proteins necessary to simulate the formation of acquired pellicle, thus preventing the assessment of its protective effect against erosive wear. Additionally, the study did not evaluate erosion associated with abrasion, which means the impact of mechanical brushing on dentin wear could not be examined. Therefore, further studies employing different methodologies are needed to investigate the preventive effects of JSG gels on the progression of dentin erosion.

The JSG gels, while showing similar results to the positive control, did not exhibit superior efficacy at the tested concentrations. Consequently, stannous fluoride remains a viable option for the treatment of erosive tooth wear.
